# The cost-effectiveness of interventions targeting lifestyle change for the prevention of diabetes in a Swedish primary care and community based prevention program

**DOI:** 10.1007/s10198-016-0851-9

**Published:** 2016-12-02

**Authors:** Anne Neumann, Lars Lindholm, Margareta Norberg, Olaf Schoffer, Stefanie J. Klug, Fredrik Norström

**Affiliations:** 1 Epidemiology and Global Health, Department of Public Health and Clinical Medicine, Umeå University, 901 87 Umeå, Sweden; 2 Center of Evidence-Based Healthcare, University Hospital, Technische Universität Dresden, Fetscherstr. 74, 01307 Dresden, Germany; 3 Cancer Epidemiology, University Cancer Center, University Hospital, Technische Universität Dresden, Fetscherstr. 74, 01307 Dresden, Germany

**Keywords:** Markov model, Cost-effectiveness, Diabetes, Prevention, Lifestyle change, I1 (Health)

## Abstract

**Background:**

Policymakers need to know the cost-effectiveness of interventions to prevent type 2 diabetes (T2D). The objective of this study was to estimate the cost-effectiveness of a T2D prevention initiative targeting weight reduction, increased physical activity and healthier diet in persons in pre-diabetic states by comparing a hypothetical intervention versus no intervention in a Swedish setting.

**Methods:**

A Markov model was used to study the cost-effectiveness of a T2D prevention program based on lifestyle change versus a control group where no prevention was applied. Analyses were done deterministically and probabilistically based on Monte Carlo simulation for six different scenarios defined by sex and age groups (30, 50, 70 years). Cost and quality adjusted life year (QALY) differences between no intervention and intervention and incremental cost-effectiveness ratios (ICERs) were estimated and visualized in cost-effectiveness planes (CE planes) and cost-effectiveness acceptability curves (CEA curves).

**Results:**

All ICERs were cost-effective and ranged from 3833 €/QALY gained (women, 30 years) to 9215 €/QALY gained (men, 70 years). The CEA curves showed that the probability of the intervention being cost-effective at the threshold value of 50,000 € per QALY gained was very high for all scenarios ranging from 85.0 to 91.1%.

**Discussion/conclusion:**

The prevention or the delay of the onset of T2D is feasible and cost-effective. A small investment in healthy lifestyle with change in physical activity and diet together with weight loss are very likely to be cost-effective.

**Electronic supplementary material:**

The online version of this article (doi:10.1007/s10198-016-0851-9) contains supplementary material, which is available to authorized users.

## Background

### Diabetes

Diabetes poses a huge burden on society both in human suffering and health expenditure terms. Diabetes is a common and costly disease that is expected to even continue to grow in prevalence and health expenditures over the coming decades. The International Diabetes Federation (IDF) estimated that approximately 52 million adults (20–79 years of age) had diabetes in the IDF EUR Region with a prevalence of 7.9% in 2014 [1]. In Sweden, the estimated number of cases was 426,800 among adults in 2014, amounting to a prevalence of 6.1% [1]. The number of diabetes-related deaths among Swedish adults was estimated at 2930 while the cost per person with diabetes in Sweden was 6310 USD [1]. It is estimated that incidence and prevalence of diabetes and consequently its costs in Sweden and Europe will increase in the future making prevention initiatives essential. An epidemiological model based on German data confirmed that unless enormous efforts are conducted into prevention programs, the number of persons with diabetes are expected to increase in the next two decades [2].

The three main types of diabetes are gestational diabetes, type 1 diabetes and type 2 diabetes (T2D); we focus on T2D in this analysis. T2D is the most common diabetes type and is characterized by insulin resistance and relative insulin deficiency. T2D develops over a long time period and is often undetected over years. During this time, people almost always first develop any of the pre-diabetic states, i.e., impaired fasting glucose (IFG), impaired glucose tolerance (IGT) or a combination of both (IFG and IGT). IFG is largely associated with an impaired insulin secretion and impaired suppression of hepatic glucose output, while IGT is mainly associated with muscle insulin resistance and defective insulin secretion [3].

### Markov models and diabetes prevention

Markov models are used to estimate future costs and effects of treatment options. They extrapolate current knowledge to be able to make decisions that have impacts on the future. Markov models may help to optimize the selection of individuals eligible for a focused intervention, such as specific lifestyle interventions [4].

Several trials have shown that prevention of T2D among individuals at high risk through lifestyle change is possible, effective and cost-effective, especially targeting diet and exercise to reduce weight [5–11]. Lifestyle modification is considered the first choice of intervention for T2D prevention as it has a good cost- and treatment-effectiveness [12]. However, the long-term cost-effectiveness of a lifestyle intervention program in a Swedish setting is still not clear. On the other hand, policymakers need to know the cost-effectiveness of interventions to prevent T2D before implementing them at population level [13].

Various studies have estimated the effectiveness and cost-effectiveness of T2D prevention initiatives starting in the increased risk state IGT [14–18]. Some studies focused on the effect of screening for T2D [18, 19]. Other studies have investigated the cost-effectiveness of healthy individuals (NGT) but only IGT as pre-diabetic state [20, 21]. However, no study to our knowledge has estimated the cost-effectiveness of T2D prevention strategies from a population-based perspective, including healthy individuals and also considering IFG and IGT as two distinct pre-diabetic states. Previous research has, nonetheless, shown that IFG and IGT are different regarding risk of developing a worsened health state and quality of life [22–25]. Norris and colleagues consequently underlined that further research is needed to define the duration of the pre-diabetes phase and to identify measurable risk factors for progression to T2D and its complications [26].

### Objective

The objective of this study was to estimate the cost-effectiveness of a T2D prevention initiative targeting weight reduction, increased physical activity and healthier diet in persons in pre-diabetic states by comparing a hypothetical intervention comparable to the Finish Diabetes Prevention Study (DPS) versus no intervention in a Swedish setting.

## Methods

A Markov model was used to study the cost-effectiveness of a hypothetical T2D prevention program based on lifestyle change through increased physical activity, healthier diet and weight reduction versus a control group where no prevention was applied. The hypothetical intervention is based on results by other T2D prevention programs targeting lifestyle change, including identification of individuals at higher risk, offering an educational course and follow-up mentoring and visits [5, 6, 20, 27, 28].

### The model

**Fig. 1:**
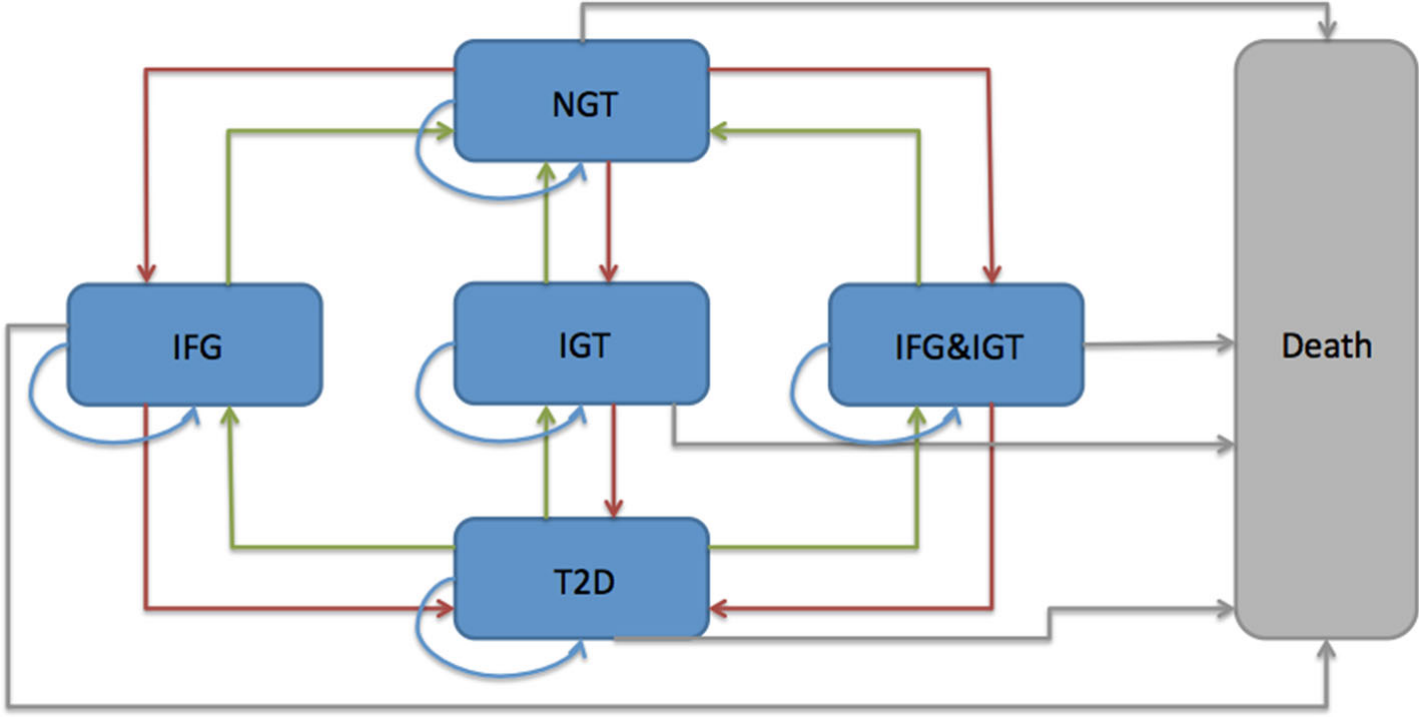
Markov model. *NGT* normal glucose tolerance, *IFG* impaired fasting glucose, *IGT* impaired glucose tolerance, *T2D* type 2 diabetes mellitus

The model consisted of six different, mutually exclusive states (Fig. 1). The states described the possible development of T2D through a simulation of hypothetical persons using average values of input parameters in combination with assumed distributions. The Markov model is characterized by a population with mean values for risk factors that are adjusted by the intervention effect.

A hypothetical person in a healthy state, NGT, could develop IFG, IGT or IFG and IGT, the pre-diabetic states. From any of the pre-diabetic states, the person could develop T2D. In return, a person with T2D could move to a pre-diabetic state or they could move from a pre-diabetic state to NGT. Direct changes between the pre-diabetic states were not possible in the model. Even though such changes are plausible, they have not been estimated in this model for simplicity reasons and because the available number of individuals for estimating the risk equation was not sufficient to build robust estimates stratified by risk factors.

The transition to move back from T2D to any pre-diabetic state was kept in the model, even though a person who once entered the T2D state is not comparable with the one who never had T2D. On the other hand, this change is possible and lifestyle interventions could have an impact on this transition. In addition, some people who had once been diagnosed with T2D might attain healthy values and will not have the same costs and complications as an average person with T2D.

At any time, the person could die and consequently move to the absorbing state death.

The length of one cycle was 1 year. A lifetime horizon was applied. For cost estimation, the societal perspective was applied.

### Input parameters

#### Transition probabilities

The risk of moving from one state to another was described by transition probabilities. The arrows indicated all possible transitions between the states during a 1-year cycle length (Fig. 1). As it was assumed that 1 year was too short to develop T2D directly from NGT, this transition was not possible. Hence, all hypothetical persons must have developed any of the three pre-diabetic states before the development of T2D. The transition probabilities in this study were extracted from a previous study based on the Västerbotten Intervention Program (VIP) [22] and they depend on the following risk factors: sex, age, education, triglyceride, blood pressure, body mass index (BMI), smoking, physical activity, snus use, nutrition, marital status, family history of T2D and self-reported health status (Table 1). In the model, all those risk factors could be adjusted according to the lifestyle of the individual. The transition probabilities were estimated based on the logistic regression of transitions in the VIP by estimating the risk of changing to a different glucose state depending on a range of risk factors [22] (Table 2).

**Table 1:** Model characteristics, modified after [15]

Characteristic	Options	Description
Sex	Male	
	Female	
Age	Metric	
Education	High	University or education >12 years education
	Middle	10–12 years of education
	Low	Compulsory school or <10 years of education
Triglyceride	Normal	<1.7 mmol/l
	High	≥1.7 mmol/l
Blood pressure	Normal	Systolic blood pressure <140 mmHg and diastolic blood pressure <90 mmHg AND no self-reported anti-hypertensive drug
	High	Self-reported anti-hypertensive drug OR systolic blood pressure ≥140 mmHg OR diastolic blood pressure ≥90 mmHg
BMI	Underweight	<18.5 kg/m^2^
	Normal	18.5–24.9 kg/m^2^
	Overweight	25–29.9 kg/m^2^
	Obese	≥30 kg/m^2^
Smoking	Never Formerly Present	
Physical activity	Physically active	Exercise at least 2–3 times/week or walk and/or cycle more than 3 times/week during leisure time or walk or cycle to work more than 5 km per way
	Moderately active	Exercise now and then but not regularly or cycle and/or walk during their leisure time at least 2–3 times per week or cycle and/or walk to work 2–5 km each way
	Sedentary	Never exercise or walk and/or cycle during their leisure time less than 2–3 times per week or take bus or car to work or cycle and/or walk to work less than 2 km per way.
Current snus use	No	Snus is an oral non-smoking tobacco that is commonly used in Sweden. It is put into the mouth, usually underneath the upper lip.
	≤4 cans per week	
	>4 cans per week	
Nutrition	At least 5 a day	At least five portions of fruits/vegetables per day
	Less than 5 a day	Less than five portions of fruits/vegetables per day
Marital status	Partnership	Married or living with spouse
	Single	Not married or widowed or divorced
Family history of T2D	Yes	Parents or siblings with T2D
	No	No parents or sibling with T2D
Self-perceived health	Good	Very good, pretty good, somewhat good
	Bad	Pretty bad, bad

*BMI* body mass index, calculation: (weight in kg)/(height in m)

**Table 2:** Risk equations for the change between disease states, adapted from [15]

From state A to state B	Regression model
NGT to IFG	Logit P(NGT to IFG) = –3.07 to 0.29 * sex + 0.01 * age + 0.11 * education + 0.19 * family history of T2D –0.09 * current snus use + 0.15 * triglyceride + 0.12 * blood pressure + 0.16 * BMI + 0.23 * smoking
NGT to IGT	Logit P(NGT to IGT) = –7.29 + 0.51 * sex + 0.06 * age + 0.09 * education + 0.14 * physical activity + 0.15 * family history of T2D + 0.41 * triglyceride + 0.34 * blood pressure + 0.21 * BMI –0.12 * smoking
NGT to IFG and IGT	Logit P(NGT to IFG and IGT) = –6.20 to 0.16 * sex + 0.04 * age –0.24 * current snus use + 0.32 * family history of T2D + 0.15 * physical activity + 0.20 * triglyceride + 0.60 * blood pressure + 0.50 * BMI –0.33 * nutrition
IFG to NGT	Logit P(IFG to NGT) = = 2.84–0.43 * family history of T2D –0.02 * age –0.23 education –0.25 * blood pressure –0.38 * BMI –0.26 * triglyceride
IGT to NGT	Logit P(IGT to NGT) = 3.06 + 0.72 * sex –0.07 * age –0.23 * education –0.37 * BMI –0.29 * self-perceived health –0.49 * triglyceride
IFG and IGT to NGT	Logit P(IFG and IGT to NGT) = –0.61 + 0.81 * sex + 1.15 * current snus use –1.24 * smoking 1 –0.77 * BMI
IFG to T2D	Logit P(IFG to T2D) = –6.98 to 0.44 * sex + 0.05 * age + 0.48 * family history of T2D + 0.36 * marital status + 0.51 * nutrition + 0.30 * triglyceride + 0.61 * blood pressure + 0.57 * BMI
IGT to T2D	Logit P(IGT to T2D) = –6.54 to 0.75 * sex + 0.07 * age + 0.87 * nutrition + 0.35 * BMI + 0.35 * blood pressure + 0.56 * triglyceride
IFG and IGT to T2D	Logit P(IFG and IGT to T2D) = - 0.55 - 0.61 * sex + 0.71 * BMI + 0.87 * family history of T2D - 1.06 * nutrition + 0.42 * smoking
T2D to IFG	Logit P(T2D to IFG) = –0.34 to 0.57* BMI - 0.68 * blood pressure –0.385 * smoking
T2D to IGT	Logit P(T2D to IGT) = –3.37 to 1.42 * family history of T2D + 1.13 self-perceived health
T2D to IFG and GT	Logit P(T2D to IFG and IGT) = –6.80 + 1.22 * sex + 1.49 * physical activity + 0.63 * education –1.07 * self-perceived health

The characteristics of the population without the intervention were based on the distribution of the characteristics in the VIP population at first examination with any of the pre-diabetic states [22] (Table 3). The success of the intervention was defined by changes in three characteristics, i.e., BMI, physical activity and nutrition, at the starting year: a weight reduction of 5% [29], an increase in physical activity by 13% [30] and an increase by 10% of the proportion of individuals consuming at least five portions of fruits and vegetables per day [6]. Changes in all three categories are well described as realistic changes due to lifestyle intervention programs.

**Table 3:** Assumed lifestyle change, effects at starting year of Markov model, no intervention vs. intervention

Characteristic	No intervention [22]	Intervention
Sex	Male	Male
	Female	Female
Age	30	30
	50	50
	70	70
Education (%)		
High	25.5	As no intervention
Middle	53.8	
Low	20.7	
Triglyceride (%)		
Normal	71.2	As no intervention
High	28.8	
Blood pressure (%)		
Normal	65.0	As no intervention
High	35.0	
BMI (%)		
Underweight	0.5	0.5
Normal	38.8	41.0^a^
Overweight	43.9	42.6^b^
Obese	16.8	16.0^c^
Smoking (%)		
Never	43.4	As no intervention
Formerly	31.1	
Present	25.5	
Physical activity (%)		
Active	9.7	18.6
Moderately	68.4	62.4
Sedentary	25.9	19.0
Current snus use (%)		
No	87.6	As no intervention
≤4 cans/week	9.3	
>4 cans/week	3.1	
Nutrition (%)		
At least	9.2	18.3
Less than	90.8	81.7
Marital status (%)		
Married	84.1	As no intervention
Single	15.9	
Family history of T2D (%)		
No	77.4	As no intervention
Yes	22.6	
Self-perceived health (%)		
Good	73.8	As no intervention
Bad	26.2	

*BMI* body mass index, calculation: (weight in kg)/(height in m)

^a^+5% from overweight with no intervention

^b^+5% from obese with no intervention, –5% to normal weight compared to no intervention

^c^–5% to overweight compared to no intervention

Results were stratified by sex and age [younger age (30 years), middle age (50 years) and older age (70 years)] at the start of the intervention.

To be conservative, it was assumed that the effectiveness of the lifestyle program decreased over time. The Finnish Diabetes Prevention Study (DPS) found that after discontinuation of active counseling, the lifestyle intervention group still had a lower incidence of T2D after 7 years compared to the no-intervention group [30]. Consequently, the effect of the intervention in this model was assumed to remain for only 7 years with a linear decrease over the 7 years to zero effectiveness in year eight and onwards. This means that after 7 years, there was no difference in transition probabilities of intervention versus no intervention. This assumed effectiveness is conservative, since a study based on the Diabetes Prevention Program (DPP) in the United States even estimated that incidence remained lower in the lifestyle group even after 10 years [27].

As the intervention targets persons at higher risk of developing T2D, all hypothetical persons start in any of the pre-diabetic states. In the starting year, 66.2, 27.2 and 6.6% of the hypothetical persons in the model had IFG, IGT or IFG and IGT, respectively, based on the distribution of participants at first examination in the VIP in the years between 1990 and 1999 [22].

#### Mortality

Age-based all-cause mortality and mortality due to T2D (ICD10: E10) in Västerbotten were taken from Statistics Sweden and the National Board of Health and Welfare based on the years 2003–2009 [31]. Five-year age ranges were estimated. The range 2003–2009 was used because data for transition probabilities were based on data from 1990 to 2009, and data for health utility weights (HUWs, see below) were based on data from 2003 to 2012. The overlapping time period is 2003–2009.

No increased risk of dying due to any of the pre-diabetic states was assumed.

#### Utilities

The self-perceived preference of one state over another is expressed by health utility weights (HUWs). A person with a HUW of zero considers his or her wellbeing equivalent to death while someone with a HUW of one considers his or her wellbeing equivalent to “perfect health”. HUWs were used to calculate quality adjusted life years (QALYs) by combining HUWs with the lengths stayed in the state. The HUWs in this study were based on the VIP population between the years 2003 and 2012 and were adjustable by age, sex, education and BMI (Table 1). The HUWs were derived from the Short-Form 36 (SF-36) questionnaire in the VIP study and translated to Short-Form 6D (SF-6D) values [32]. Beta regression was used to estimate HUWs based on the defined risk factors and stratified by glucose group [23]. The estimated HUWs with the assumed effects (Table 2) indicate only minor changes between ages and sexes (Table 4). HUWs decrease with worsened glucose state, with the exception of IFG and IGT. Women had lower HUWs compared to men. Older age (70 years) leads to a very minor decrease in HUWs, with the exception of IFG and IGT. As for transition probabilities described above, the effect of the intervention was assumed to remain for only 7 years with a linear decrease of the effectiveness to zero in year eight. This means that after 7 years, there was no difference in HUWs of intervention versus no intervention (HUWs not shown).

**Table 4:** Health utility weight by sex, age and intervention, based on population of Västerbotten Intervention Program (2003–2012)

HUW	No intervention						Intervention				
Sex/age	NGT	IFG	IGT		IFG and IGT	T2D	NGT	IFG	IGT	IFG and IGT	T2D
Male/30	0.83	0.82	0.73		0.77	0.72	0.83	0.82	0.73	0.77	0.72
Male/50	0.83	0.82	0.72		0.79	0.72	0.83	0.82	0.72	0.79	0.72
Male/70	0.83	0.81	0.72		0.81	0.72	0.83	0.82	0.72	0.81	0.72
Female/30	0.80	0.79	0.70		0.74	0.68	0.80	0.79	0.70	0.74	0.68
Female/50	0.80	0.79	0.70		0.76	0.68	0.80	0.79	0.70	0.76	0.68
Female/70	0.80	0.78	0.69		0.78	0.68	0.80	0.78	0.69	0.78	0.68

*HUW* health utility weight

#### Costs

Cost for T2D was divided into direct (inpatient care, outpatient hospital care, outpatient primary care, antidiabetic drugs, antihypertensive drugs, lipid-lowering drugs, devices) and indirect (sickness absence, early retirement, production loss due to mortality) costs (Table 5) [33, 34]. The direct annual cost due to T2D in Sweden was estimated at 3602 € [33]. It was estimated that 57% of the total cost was due to indirect costs [34]. Therefore, the total annual cost for T2D was estimated at 8376.74 €. Several studies estimated that IGT, IFG and IFG and IGT consume a substantially higher amount of resources compared to NGT [35–37]. Palmer and colleagues assumed a cost of 46% of T2D cost [37]. NGT had no costs associated compared to the other states.

**Table 5:** Annual cost of glucose states, from published data

State	Cost (euros)	Source
NGT	0.00	
IFG	3853.30	46% of T2D total cost [35–37]
IGT	3853.30	46% of T2D total cost [35–37]
IFG and IGT	3853.30	46% of T2D total cost [35–37]
T2D	8376.74	direct (3602.00 €) + indirect (4774.74 €) costs
Direct costs	3602.00	Mean direct costs of T2D per patient, year 2004, year 2007, euros, including: inpatient care, outpatient hospital care; outpatient primary care, antidiabetic drugs, antihypertensive drugs, lipid-lowering drugs, devices; SD: 9537 € [33]
Indirect costs	4774.74	57% of total T2D cost; including: sickness absence, early retirement, production loss due to mortality [34]

*NGT* normal glucose state

*IFG* impaired fasting glucose

*IGT* impaired glucose state

*IFG and IGT* combination of IFG and IGT

*T2D* type 2 diabetes mellitus

#### Intervention cost

The cost for the lifestyle intervention was taken from a low-intensive population-based intervention in Germany, the Saxon Diabetes Prevention Program [20]. The intervention consists of three steps, i.e., firstly identifying individuals at higher risk for developing T2D, secondly conducting weekly courses for 8 weeks on the physiology of the body, healthy eating, exercise and motivation guided by prevention managers and thirdly offering follow-up mentoring with the prevention manager which continues as long as the participant wishes. Prevention managers are individuals trained for motivational counseling and diabetes prevention (diet and physical activity). Cost for the 1st year was estimated to be 390.43 €, while cost for years two to five were 189.93 €. The intervention cost includes the cost for screening (1st year), a course on healthy lifestyle (1st year) and follow-up through trained prevention managers (2nd to 5th years). The estimations were based on a cost analysis of the Saxon Diabetes Prevention Program from a societal perspective [20]. Costs for the intervention occur only for the first 5 years.

All costs and quality adjusted life years (QALYs) were discounted at 3% according to the Guidelines of the International Society for Pharmacoeconomics and Outcome Research (ISPOR) Sweden [38].

### Analyses

First, analyses were done deterministically using all input parameters as fixed values. Deterministic modeling always returns the same results using the same input parameters.

Second, analyses were conducted probabilistically based on Monte Carlo simulation using defined statistical distributions of input parameters to simulate results. Probabilistic modeling was used to investigate the impact of statistical scattering of the input parameters on the model results, which elicits the uncertainty of the results. One thousand replicates were performed based on underlying probability distributions for input parameters and the mean of the costs and QALYs was calculated. Percentile confidence intervals were estimated for cost and QALYs. To estimate the percentile confidence intervals, the 2.5th and 97.5th ordered values were used as lower and upper limits of the confidence interval, respectively.

Six hypothetical scenarios were run, for men and women at younger (30), middle (50) and older (70) age, to describe the cost-effectiveness of the intervention for both sexes at different age groups. Cost and QALY differences between no intervention and intervention, and incremental cost-effectiveness ratios (ICERs, cost per QALY gained) were estimated and visualized in the cost-effectiveness quadrants (cost-effectiveness planes, CE planes) as scatterplots of the Monte Carlo simulation results. The cost-effectiveness acceptability curves (CEA curves) displayed the probability that the data are consistent with a true cost-effectiveness ratio falling below a specified threshold value. CE planes and CEA curves were estimated for all six sex-age scenarios to enhance readability of model results. Cost-effectiveness planes and cost-effectiveness acceptability curves assist in reading the distribution of the probabilistic outcomes and the probability of whether the intervention is cost-effective assuming different threshold values.

#### Distribution of probability parameters for probabilistic analysis

A beta distribution was chosen for transition probabilities and HUWs, since the beta distribution is appropriate for modeling values between 0 and 1. The method of moments was applied [39].

A gamma distribution was chosen for cost parameters. A gamma distribution is recommended for cost estimations since cost data are constrained to be non-negative and are made up of counts of resource use weighted by unit costs [39].

As no standard deviation was provided in the sources used for the cost parameters, the mean values of the cost estimations were assumed for the standard deviations. This is a commonly applied assumption for the gamma distribution in probabilistic analyses using the specific characteristics of the gamma distribution [39].

The assumed threshold for cost-effectiveness was 50,000 € per QALY gained. This means that all interventions that cost less than 50,000 € per one QALY gained through intervention were considered as cost-effective. This arbitrary threshold is used in other cost-effectiveness analyses [40]. However, cost-effectiveness acceptability curves have been presented to describe the cost-effectiveness assuming different threshold values.

#### Sensitivity analyses

Sensitivity analyses were conducted in five ways:

- Probabilistic sensitivity analysis was performed with Monte Carlo model simulations with 1000 replications to account for uncertainty in multiple parameters (probabilistic).
- The possibility of changing from T2D to any of the pre-diabetic states was removed to investigate the influence of this transition on the model results (deterministic).
- The cost in states and the cost of the intervention were varied by plus and minus 10% to investigate the influence of the variance on cost estimates and because cost estimates revealed greatest uncertainty (deterministic).
- The time the intervention showed effect was doubled from 7 years to 14 years to investigate whether this lower assumpted duration of effect influenced the results.
- The reduction in weight was changed from 5 to 3.3%, which corresponds to the average decreased weight in community-based weight loss programs in the United States (Table 2, [41]) to investigate the influence of less optimistic effects (deterministic). Further, the reduction in weight of 3.3% was assumed to be the only effect (deterministic).

## Results

### Deterministic analyses

Running the model deterministically (stable input parameters) with different scenarios of age and sex showed the effect that the intervention was more costly than no intervention but also gained higher QALYs (Table 6). The higher QALYs for the intervention group showed that the intervention was effective compared to no intervention. The ICER was highest for men at older age (men, age 70: 9215 € per QALY gained) and lowest for women at younger age (women, age 30: 3833 € per QALY gained). The ICER was more favorable for women compared to men and for younger age compared with older age (Table 6). All ICERs were cost-effective assuming a cost-effectiveness threshold of 50,000 € per QALY gained.

**Table 6:** Cost, QALY and ICER, deterministic

	Male			Female		
	Cost	QALY	ICER	Cost	QALY	ICER
Age 30						
No intervention	53,915	19.9		46,037	20.1	
Intervention	56,383	20.5	4109	48,355	20.7	3833
Age 50						
No intervention	65,013	14.5		59,742	15.2	
Intervention	67,690	14.9	6088	62,341	15.6	5656
Age 70						
No intervention	44,721	7.5		47,739	8.3	
Intervention	46,819	7.7	9215	49,957	8.6	8728

*QALY* quality adjusted life year, *ICER* incremental cost-effectiveness ratio, change in cost/change in effect, euros per 1 QALY gained

### Probabilistic analyses

Running the model probabilistically 1000 times while assuming specified statistical distributions elicits the uncertainty of the results. The mean cost difference was higher than the median cost difference for all scenarios, while the difference in effect between mean and median values was very small (Table 7). The difference between mean and median in cost was due to the right skewed distribution of the data. The percentile interval for the cost difference was large, ranging from cost-saving results to higher cost for the intervention (Table 7). This large range derived from the large assumed standard deviation of the cost, i.e., standard deviation being equal to the mean value of the costs. The ICERs derived by probabilistic analysis were comparable to the deterministic results (Tables 6, 7).

**Table 7:** Incremental cost and quality adjusted life year (QALY), intervention vs no intervention, probabilistic, 1000 simulations

	Male			Female		
	Δ Cost	Δ QALY	ICER	Δ Cost	Δ QALY	ICER
Age 30						
Mean	3228	0.60	5 402	2117	0.61	3455
Median	2592	0.59		1783	0.61	
Percentile interval	–33,852; 41,414	0.27; 0.97		–30,122; 34,400	0.29; 0.97	
Age 50						
Mean	2983	0.43	6 885	2065	0.45	4552
Median	2392	0.43		1962	0.45	
Percentile interval	–35,499; 37,372	0.16; 0.76		–30,525; 37,260	0.12; 0.81	
Age 70						
Mean	2138	0.22	9 505	2192	0.25	8771
Median	1866	0.22		1 995	0.25	
Percentile interval	–11,322; 17,653	0.12; 0.35		–12,624; 19,436	0.13; 0.40	

*QALY* quality adjusted life year, *ICER* incremental cost-effectiveness ratio

#### Cost-effectiveness planes

**Fig. 2:**
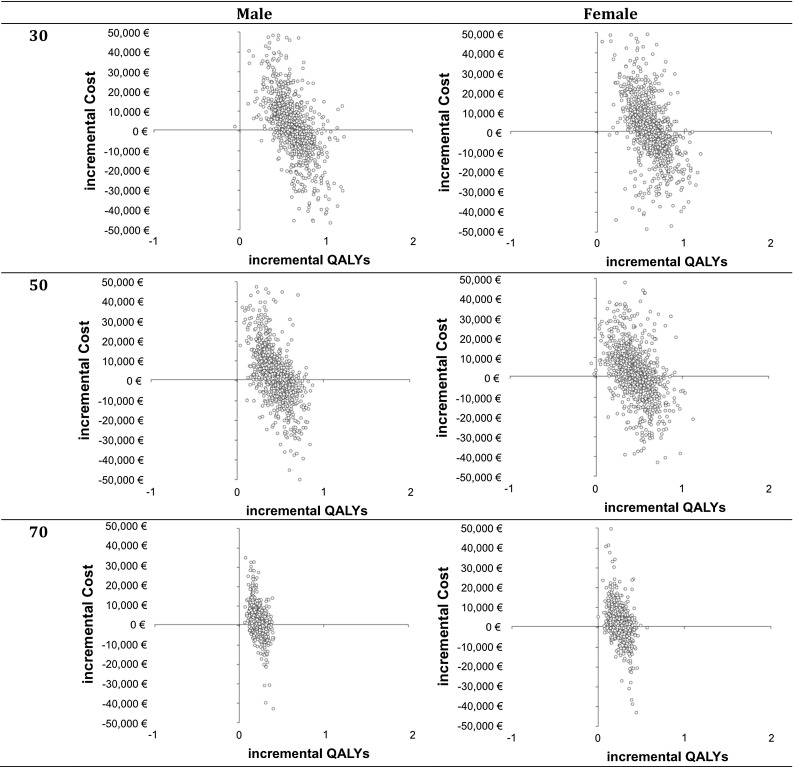
Cost-effectiveness planes by age [younger (30), middle (50), older (70)] and sex (male, female), 1000 simulations

The cost-effectiveness planes (CE planes) showed the distribution of results according to the Monte Carlo simulation of the probabilistic analyses (Fig. 2). All CE planes indicated that the intervention almost always increased QALYs. The intervention partly saved costs and incurred costs; however, the range in cost difference was large as indicated by the percentile range above (Table 7). The spread of the incremental QALYs decreased with age.

#### Cost-effectiveness acceptability curves (CEA curves)

**Fig. 3:**
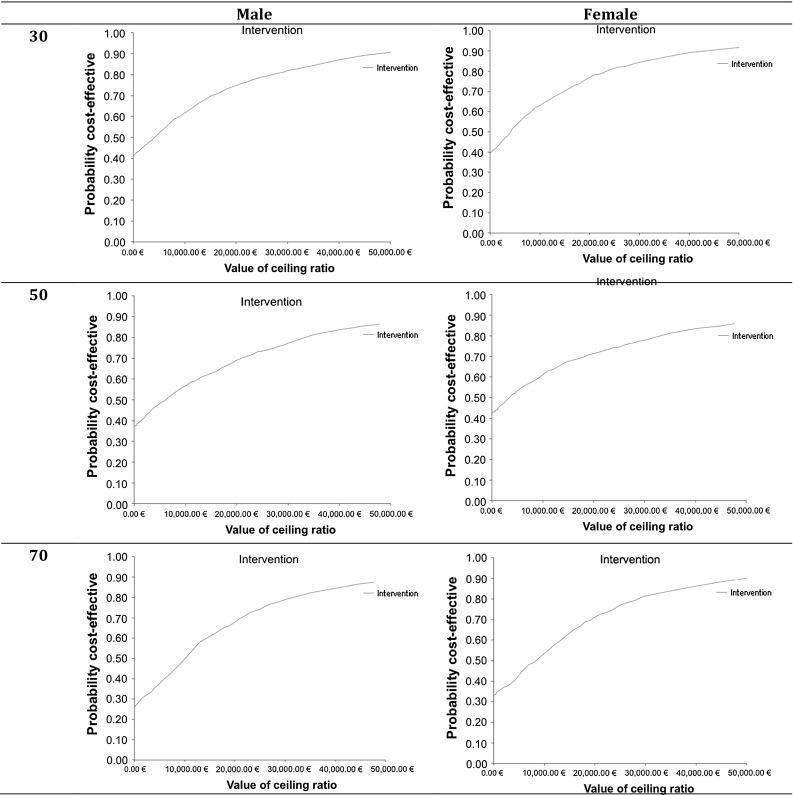
Cost-effectiveness acceptability curves by age [younger (30), middle (50), older (70)] and sex (male, female)

The cost-effectiveness acceptability curves (CEA curves) showed that the probability of the intervention being cost-effective was very high for all scenarios for the threshold value of 50,000 € per QALY gained (Fig. 3). The probability of being cost-effective at the threshold value of 50,000 € per QALY gained ranged from 85.0% (men, 50 years) to 91.1% (men, 30 years).

### Sensitivity analyses

- The incremental cost, QALY and ICER based on the probabilistic results (Table 7), the CE planes (Fig. 2) and the CEA curves (Fig. 3) indicated that the uncertainty around the cost distribution was high. Therefore, additional one-way sensitivity analyses were conducted [see (c)].
- Not allowing the model to transit from T2D to any pre-diabetic state influenced the ICER. While the difference between this change and the model with the transition was low among older people, the ICER got less favorable with middle age and was far from cost-effective for younger people (Table S1, Supplement). Incremental QALYs between intervention and no intervention were small for people in younger age leading to less favorable ICERs. Comparing the CE planes showed that the uncertainty around incremental cost was similar to the results of the model including the transition back from T2D (Fig. S1, Supplement). However, the spread among incremental QALYs was higher among younger age in the model without the transition (Fig. S1, Supplement).
- Modifying the cost in the states and the cost of the intervention by ±10% did not have a huge impact on the ICER (Tables S2–S5, Supplement).
- Doubling the time the intervention showed effect (with linear decrease over time), which influenced the transition probabilities and the HUWs in the intervention, did not have a huge effect on the ICER (Table S6, Supplement).
- Adjusting the assumed effect of the intervention to lower effects in weight change (Table S7; Fig. S2, Supplement) or lower effects in weight change and no other effects (Table S8; Fig. S3, Supplement) did not have a huge impact on the ICER.

## Discussion

### Principal findings

The results of this analysis showed that the described hypothetical intervention was very cost-effective for men and women at all ages. The cost-effectiveness planes showed the distribution of the cost and outcomes and the cost-effectiveness acceptability curves underlined that the probability that the intervention was cost-effective was high assuming a threshold value of 50,000 € per QALY gained. Assuming that the described hypothetical intervention and its effect was similar to those of the DPS, this analysis showed what would happen if a DPS-like intervention were implemented in Västerbotten.

### Other studies

Several studies have estimated the cost-effectiveness of lifestyle intervention. However, none focused on the costs and effects of NGT, IFG, IGT, and IFG and IGT on the development of T2D. In addition, it is difficult to compare the results of different cost-effectiveness studies with each other as methods, health care systems, costs included, perspectives, lifestyle interventions and other factors differ [13]. Nonetheless, in the following, the results of this study were compared with other modeling studies focusing on T2D prevention through lifestyle change.

Similar lifestyle interventions for preventing T2D were also cost-effective [14, 15, 19, 20, 42], cost-saving [16–18, 21, 43, 44] or had mixed results [45].

A Markov Monte Carlo simulation on the cost-effectiveness of screening for T2D found that early detection and prevention of T2D by either lifestyle intervention or metformin may be cost-effective, with 562.54 € per QALY for lifestyle intervention [19]. Forster and colleagues analyzed the cost-effectiveness of two diet and exercise interventions, showing that those interventions have the potential to be cost-effective [42]. Smith and colleagues estimated that the DPP-like intervention cost less than $20,000 per QALY gained in approximately 78% and less than $50,000 per QALY gained in approximately 86% of model iterations using cost-effectiveness acceptability curves [14]. An estimation of the cost-effectiveness of the DPP showed that the intervention could provide a financial return on investment for private payers and long-term benefits for Medicare with an estimated cost of $1288 per QALY gained at age 50 [15]. A modeling study based on an example from Germany estimated that lifestyle interventions for primary prevention of T2D have a high potential to be cost-effective [20].

In a modeling study examining the effect of lifestyle intervention for preventing T2D in an Australian setting, the program was dominant over no treatment (cost- and life-saving) [21]. Häussler and Breyer estimated that a German lifestyle intervention to reduce obesity among obese people without increased glucose tolerance can pay for itself from the point of view of a health insurer [43]. The evaluation of the European Community-based project “10,000 Steps Ghent” showed that the intervention, which focused on increased physical activity, was dominant compared to no intervention [44]. A cost-effectiveness evaluation of the Diabetes Prevention Program Outcomes Study estimated that lifestyle intervention among people with pre-diabetes produces long-term social benefits which exceed intervention costs [16]. A simulation model based on Swedish data estimated that lifestyle intervention programs save a mean total of 1853 € per patient [17]. Liu and colleagues estimated that programs to prevent T2D using lifestyle interventions were cost-saving in a Chinese setting [18].

### Strengths and weaknesses

This study has several strengths. Both the health utility weight and the risk equations that define the transition probabilities were estimated from the same source, the Västerbotten Intervention Program (VIP) and for NGT, IFG, IGT, and T2D. As other studies only included IGT instead of IFG and IGT, and very few studies considered NGT, this analysis provides essential information on the prevention of T2D and its pre-states. Several sensitivity analyses including probabilistic sensitivity analysis enable the reader to judge the soundness of the model and its input parameters. In addition, CEA curves and CE planes assist the reader in judging the stability of the results and the change with assumed threshold value.

On the other hand, some limitations are to be mentioned. As the focus of this study was the prevention of T2D, this model did not differentiate between T2D with and without complications. However, neither complications nor prevention of complications were in the focus of this analysis. Hypothetical persons who just entered the T2D state would be underestimated in HUW and overestimated in cost terms. Over time, the complications increase and the cost increases, while the HUWs decrease. Hypothetical persons who were in T2D for a long time would be underestimated in cost estimates and overestimated in HUWs in this model. However, as a lifetime time horizon was used here, these differences disappear or are marginal since sometimes over- and sometimes underestimations would balance using the average value. In addition, due to the lack of information on cost of IFG compared to IGT, the cost within the pre-diabetic states was assumed to be the same. Further, data for transition probabilities and health utility weights for T2D with or without complications were not available. However, it has to be kept in mind that the cost of T2D is strongly associated with complications, while the combination of micro- and macrovascular complications incurs the highest cost [45, 46]. Further, as the population of inhabitants in Västerbotten is fairly small (approximately 260,000), the robustness of the mortality values could be questioned. However, cross-checking the mortality in Västerbotten with the mortality in entire Sweden showed no significant difference. As input parameters for the model were taken from different sources, this could limit the internal validity of the model. However, it was impossible to extract all necessary information for the model from the same source. Nevertheless, most data were taken from the Västerbotten population or the Diabetes Prevention Study in Finland. In addition, the internal validity of the model could be reduced as different studies were used to describe the effect of the intervention. Further, IGT was associated with lower HUWs compared to IFG and IGT (Table 4), which seems counter-intuitive. Having IGT in the described risk profile (Table 3) seems to reveal worse HUWs for IGT compared to IFG and IGT. Nonetheless, the HUWs of all pre-diabetic states ranged between NGT and T2D and the differences were too small to have huge impacts on the final results.

### Importance of assumptions and model structure

All models are based on a certain number of assumptions, as not all data are available. Some assumptions in this model were based on data of the VIP and some assumptions on results from other studies. The main assumptions were: the cost of all pre-diabetic states was the same and was 46% of those of T2D; the development of pre-diabetic states after T2D was possible; the effect of the intervention only lasted for 7 years, with a linear decrease over time; the effect of lifestyle change was according to the Diabetes Prevention Study; there was no increased risk of dying due to pre-diabetic states; the chosen statistical distributions for the probabilistic estimations were used; and the standard deviation equaled the mean cost. The lack of data is indeed one of the reasons modeling is needed. It is recognized that modeling studies are valuable in the decision-making process where real-life data or intervention effectiveness is insufficient or absent [21]. Therefore, informed and well-guided assumptions need to be made for decision-making.

This analysis has shown that model structure is an important issue of modeling. The possible transition between T2D and any pre-diabetic state could be argued. Markov models ignore information on what has happened in the cycles before and, therefore, do not differentiate in any pre-diabetic state between those who moved from T2D to a pre-diabetic state and those in the pre-diabetic state who never had T2D. On the other hand, this transition is technically and physiologically possible and, due to the limitation that this model does not differentiate between less severe T2D and T2D with complications, it allows separation of those people with low-profile T2D from those in more severe states. It was decided to keep this transition, as it is plausible and underlines the dynamics in the model, and to have its influence checked in a sensitivity analysis. The sensitivity analysis showed that this assumption does have a large impact on the result.

Clinical trials have largely targeted IGT, while also other trials have shown that prevention in individuals with IFG can be effective [47]. The effectiveness in our model, i.e., influence on weight, physical activity and diet, was also taken from clinical trials, which only included IGT as the pre-diabetic state. However, the results of the effectiveness in our model were translated to effects based on risk equations for transition probabilities [22] and health utility weights [23]. Therefore, the influence of IFG, IGT, and IFG and IGT were modeled separately based on distinct functions.

Possible transitions between the pre-diabetic states were not modeled, as the population used in the model was too small to lead to robust estimates. On the other hand, such transitions would not have a huge impact on the outcome of the model as both the effect (HUWs) and the costs were very similar between pre-diabetic states.

Even though QALYs gained were low, other benefits that derive from the intervention should not be omitted. Prevention of pre-diabetic conditions and T2D as well as the adoption of a healthy lifestyle also have positive impacts on other diseases such as cardiovascular diseases and some cancers. Therefore, the positive effect in terms of prevention of cost due to cardiovascular diseases and cancer of this model is, therefore, likely to be underestimated.

The intervention was assumed to only have effects on BMI, nutrition and physical activity, and only last for 7 years with a linear decrease. For some people, lifestyle intervention programs could have a longer lasting effect than just 7 years. The Chinese Da Qing study, for example, showed an effectiveness of lifestyle intervention even after 20 years [48]. However, sensitivity analyses have shown that doubling the duration of time of the intervention showed a small impact on the results, which can be explained by the highest difference in effect between intervention and no intervention being in the 1st year and thereafter decreasing substantially within a few years.

### Implications for policy makers

This analysis aimed to provide indicators for long-term allocation of healthcare resources in Sweden and other European countries. In line with other studies, the prevention of T2D through lifestyle change can be very cost-effective. Targeting weight reduction through increase of physical activity and change in diet would have a strong implication on future T2D risk. The return on investment in T2D prevention initiatives in a group setting is high. This analysis shows that implementing a DPS-like intervention in Västerbotten would have a high potential to be cost-effective.

However, one has to be aware that targeting the T2D epidemic only at individual level may likely not suffice. Interventions and actions at policy and environmental level need to supplement individual interventions for a sustainable reduction in T2D incidence [49]. The rising incidence is driven by a wide mix of factors at different levels, such as individual susceptibility, food supply, transportation use, climate change or the economic situation of the country [50]. Targeting individual interventions is one important step towards slowing the progression of T2D but should not be the only initiative.

## Conclusion

The prevention or the delay of the onset of T2D is feasible and cost-effective. A small investment in healthy lifestyle with change in physical activity and diet together with weight loss are very likely to be cost-effective. Having a lifestyle intervention comparable to DPS implemented in the north of Sweden has a high potential to be effective and cost-effective.

## Electronic supplementary material

Below is the link to the electronic supplementary material.

Supplementary material 1 (DOCX 1273 kb)

## References

[1] International Diabetes Federation: Diabetes atlas update 2014 (2014) 35914061

[2] Brinks R, Tamayo T, Kowall B, Rathmann W (2012). Prevalence of type 2 diabetes in Germany in 2040: estimates from an epidemiological model. Eur. J. Epidemiol..

[3] WHO (1999). Definition, diagnosis and classification of diabetes mellitus and its complications, part 1: diagnosis and classification of diabetes mellitus.

[4] Postmus D, de Graaf G, Hillege HL, Steyerberg EW, Buskens E (2012). A method for the early health technology assessment of novel biomarker measurement in primary prevention programs. Stat. Med..

[5] Knowler WC, Barrett-Connor E, Fowler SE, Hamman RF, Lachin JM, Walker EA, Nathan DM (2002). Reduction in the incidence of type 2 diabetes with lifestyle intervention or metformin. N. Engl. J. Med..

[6] Tuomilehto J, Lindstrom J, Eriksson JG, Valle TT, Hamalainen H, Ilanne-Parikka P, Keinanen-Kiukaanniemi S, Laakso M, Louheranta A, Rastas M (2001). Prevention of type 2 diabetes mellitus by changes in lifestyle among subjects with impaired glucose tolerance. N. Engl. J. Med..

[7] Kosaka K, Noda M, Kuzuya T (2005). Prevention of type 2 diabetes by lifestyle intervention: a Japanese trial in IGT males. Diabetes Res. Clin. Pract..

[8] Ramachandran A, Snehalatha C, Mary S, Mukesh B, Bhaskar AD, Vijay V (2006). The Indian Diabetes Prevention Programme shows that lifestyle modification and metformin prevent type 2 diabetes in Asian Indian subjects with impaired glucose tolerance (IDPP-1). Diabetologia.

[9] Eriksson KF, Lindgarde F (1991). Prevention of type 2 (non-insulin-dependent) diabetes mellitus by diet and physical exercise. The 6-year Malmo feasibility study. Diabetologia.

[10] Pan XR, Li GW, Hu YH, Wang JX, Yang WY, An ZX, Hu ZX, Lin J, Xiao JZ, Cao HB (1997). Effects of diet and exercise in preventing NIDDM in people with impaired glucose tolerance. The Da Qing IGT and Diabetes Study. Diabetes Care.

[11] Mensink M, Feskens EJ, Saris WH, De Bruin TW, Blaak EE (2003). Study on Lifestyle Intervention and Impaired Glucose Tolerance Maastricht (SLIM): preliminary results after one year. Int. J. Obes. Relat. Metab. Disord..

[12] Shin JA, Lee JH, Kim HS, Choi YH, Cho JH, Yoon KH (2012). Prevention of diabetes: a strategic approach for individual patients. Diabetes/Metab. Res. Rev..

[13] Saha S, Gerdtham UG, Johansson P (2010). Economic evaluation of lifestyle interventions for preventing diabetes and cardiovascular diseases. Int. J. Environ. Res. Pub. Health..

[14] Smith KJ, Hsu HE, Roberts MS, Kramer MK, Orchard TJ, Piatt GA, Seidel MC, Zgibor JC, Bryce CL (2010). Cost-effectiveness analysis of efforts to reduce risk of type 2 diabetes and cardiovascular disease in southwestern Pennsylvania, 2005–2007. Prev. Chronic Dis..

[15] Ackermann RT, Marrero DG, Hicks KA, Hoerger TJ, Sorensen S, Zhang P, Engelgau MM, Ratner RE, Herman WH (2006). An evaluation of cost sharing to finance a diet and physical activity intervention to prevent diabetes. Diabetes Care.

[16] Dall TM, Storm MV, Semilla AP, Wintfeld N, O’Grady M, Narayan KM (2015). Value of lifestyle intervention to prevent diabetes and sequelae. Am. J. Prev. Med..

[17] Lindgren P, Lindstrom J, Tuomilehto J, Uusitupa M, Peltonen M, Jonsson B, de Faire U, Hellenius ML (2007). Lifestyle intervention to prevent diabetes in men and women with impaired glucose tolerance is cost-effective. Int. J. Technol. Assess. Health Care.

[18] Liu X, Li C, Gong H, Cui Z, Fan L, Yu W, Zhang C, Ma J (2013). An economic evaluation for prevention of diabetes mellitus in a developing country: a modelling study. BMC Pub. Health.

[19] Schaufler TM, Wolff M (2010). Cost effectiveness of preventive screening programmes for type 2 diabetes mellitus in Germany. Appl. Health Econ. Health Policy.

[20] Neumann A, Schwarz P, Lindholm L (2011). Estimating the cost-effectiveness of lifestyle intervention programmes to prevent diabetes based on an example from Germany: Markov modelling. Cost. Eff. Resour. Alloc.: C/E.

[21] Palmer AJ, Tucker DM (2012). Cost and clinical implications of diabetes prevention in an Australian setting: a long-term modeling analysis. Prim. Care Diabetes.

[22] Neumann A, Norberg M, Schoffer O, Norström F, Johansson I, Klug SJ, Lindholm L (2013). Risk equations for the development of worsened glucose status and type 2 diabetes mellitus in a Swedish intervention program. BMC Pub. Health.

[23] Neumann A, Schoffer O, Norstrom F, Norberg M, Klug SJ, Lindholm L (2014). Health-related quality of life for pre-diabetic states and type 2 diabetes mellitus: a cross-sectional study in Vasterbotten, Sweden. Health Qual. Life Outcomes.

[24] Vaatainen S, Keinanen-Kiukaanniemi S, Saramies J, Uusitalo H, Tuomilehto J, Martikainen J (2014). Quality of life along the diabetes continuum: a cross-sectional view of health-related quality of life and general health status in middle-aged and older Finns. Qual. Life Res..

[25] Faerch K, Witte DR, Tabak AG, Perreault L, Herder C, Brunner EJ, Kivimaki M, Vistisen D (2013). Trajectories of cardiometabolic risk factors before diagnosis of three subtypes of type 2 diabetes: a post hoc analysis of the longitudinal Whitehall II cohort study. Lancet Diabetes Endocrinol..

[26] Norris SL, Kansagara D, Bougatsos C, Nygren P, Fu R: In screening for type 2 diabetes mellitus: update of 2003 systematic evidence review for the US preventive services task force. Rockville (MD); 2008: U.S. Preventive Services Task Force evidence syntheses, formerly systematic evidence reviews]

[27] Knowler WC, Fowler SE, Hamman RF, Christophi CA, Hoffman HJ, Brenneman AT, Brown-Friday JO, Goldberg R, Venditti E, Nathan DM (2009). 10-year follow-up of diabetes incidence and weight loss in the Diabetes Prevention Program Outcomes Study. Lancet.

[28] Norberg M, Wall S, Boman K, Weinehall L (2010). The Vasterbotten Intervention Programme: background, design and implications. Glob. health action.

[29] Herman WH, Edelstein SL, Ratner RE, Montez MG, Ackermann RT, Orchard TJ, Foulkes MA, Zhang P, Saudek CD, Brown MB (2013). Effectiveness and cost-effectiveness of diabetes prevention among adherent participants. Am. J. Manag. Care..

[30] Lindstrom J, Ilanne-Parikka P, Peltonen M, Aunola S, Eriksson JG, Hemio K, Hamalainen H, Harkonen P, Keinanen-Kiukaanniemi S, Laakso M (2006). Sustained reduction in the incidence of type 2 diabetes by lifestyle intervention: follow-up of the Finnish Diabetes Prevention Study. Lancet.

[31] Scialstyrelsen, The Health and Welfare Statistical Database, all cause of death and death due to ICD10: E10. http://www.socialstyrelsen.se/statistics/statisticaldatabase/causeofdeath. Assessed May 2015

[32] Brazier JE, Roberts J (2004). The estimation of a preference-based measure of health from the SF-12. Med. Care.

[33] Ringborg A, Martinell M, Stalhammar J, Yin DD, Lindgren P (2008). Resource use and costs of type 2 diabetes in Sweden—estimates from population-based register data. Int. J. Clin. Pract..

[34] Henriksson F, Jonsson B (1998). Diabetes: the cost of illness in Sweden. J. Intern. Med..

[35] Nichols GA, Glauber HS, Brown JB (2000). Type 2 diabetes: incremental medical care costs during the 8 years preceding diagnosis. Diabetes Care.

[36] American Diabetes Association (2008). Economic costs of diabetes in the US in 2007. Diabetes Care.

[37] Palmer AJ, Roze S, Valentine WJ, Spinas GA, Shaw JE, Zimmet PZ (2004). Intensive lifestyle changes or metformin in patients with impaired glucose tolerance: modeling the long-term health economic implications of the diabetes prevention program in Australia, France, Germany, Switzerland, and the United Kingdom. Clin. Ther..

[38] TPBB: General guidelines for economic evaluations from the Pharmaceutical Benefits Board (LFNAR 2003:2). 2003

[39] Briggs A, Claxton K, Mark S (2006). Decision modelling for health economic evaluation.

[40] Witt CM, Reinhold T, Jena S, Brinkhaus B, Willich SN (2009). Cost-effectiveness of acupuncture in women and men with allergic rhinitis: a randomized controlled study in usual care. Am. J. Epidemiol..

[41] Kahn R, Davidson MB (2014). The reality of type 2 diabetes prevention. Diabetes Care.

[42] Forster M, Veerman JL, Barendregt JJ, Vos T (2011). Cost-effectiveness of diet and exercise interventions to reduce overweight and obesity. Int. J. Obes..

[43] Haussler J, Breyer F (2015). Does diabetes prevention pay for itself? Evaluation of the MOBILIS program for obese persons. Eur. J. Health Econ.

[44] De Smedt D, De Cocker K, Annemans L, De Bourdeaudhuij I, Cardon G (2012). A cost-effectiveness study of the community-based intervention ‘10,000 Steps Ghent’. Pub. Health Nutr..

[45] Johansson P, Ostenson CG, Hilding AM, Andersson C, Rehnberg C, Tillgren P (2009). A cost-effectiveness analysis of a community-based diabetes prevention program in Sweden. Int. J. Technol. Assess. Health Care.

[46] Henriksson F, Agardh CD, Berne C, Bolinder J, Lonnqvist F, Stenstrom P, Ostenson CG, Jonsson B (2000). Direct medical costs for patients with type 2 diabetes in Sweden. J. Intern. Med..

[47] Saito T, Watanabe M, Nishida J, Izumi T, Omura M, Takagi T, Fukunaga R, Bandai Y, Tajima N, Nakamura Y (2011). Lifestyle modification and prevention of type 2 diabetes in overweight Japanese with impaired fasting glucose levels: a randomized controlled trial. Arch. Intern. Med..

[48] Li G, Zhang P, Wang J, Gregg EW, Yang W, Gong Q, Li H, Jiang Y, An Y, Shuai Y (2008). The long-term effect of lifestyle interventions to prevent diabetes in the China Da Qing Diabetes Prevention Study: a 20-year follow-up study. Lancet.

[49] Schwarz PE, Riemenschneider H (2016). Slowing down the progression of type 2 diabetes: we need fair, innovative, and disruptive action on environmental and policy levels!. Diabetes Care.

[50] Wareham NJ, Herman WH (2016). The clinical and public health challenges of diabetes prevention: a search for sustainable solutions. PLoS Med.

