# Supplementary material for: The cost-effectiveness of interventions targeting lifestyle change for the prevention of diabetes in a Swedish primary care and community based prevention program
Source: Eur J Health Econ. 2016 Dec 2;18(7):905–19. doi: 10.1007/s10198-016-0851-9 (PMC5533851; doi:10.1007/s10198-016-0851-9)
Supplement: Supplementary file 1 — Supplementary material 1 (DOCX 1273 kb) [file 10198_2016_851_MOESM1_ESM.docx]

**Supplement**

Title: The cost-effectiveness of interventions targeting lifestyle change for the prevention of diabetes in a Swedish primary care and community based prevention program

Journal name: The European Journal of Health Economics

Authors: Anne Neumann^1,2^, Lars Lindholm^1^, Margareta Norberg^1^, Olaf Schoffer^3^, Stefanie J Klug^3^, Fredrik Norström^1^

Affiliations:

^1^ Epidemiology and Global Health, Department of Public Health and Clinical Medicine, Umeå University, SE-901 87, Umeå, Sweden

^2^ Center of Evidence-Based Healthcare, University Hospital, Technische Universität Dresden, Fetscherstr. 74, 01307 Dresden, Germany

^3^ Cancer Epidemiology, University Cancer Center, University Hospital, Technische Universität Dresden, Fetscherstr. 74, 01307 Dresden, Germany

E-Mail address of corresponding author: Anne.Neumann@uniklinikum-dresden.de

Table S1: Sensitivity analysis: Cost, QALY and ICER, deterministic, when transition from type 2 diabetes to any pre-diabetic states was not possible

|  | **Male** | | | **Female** | | | |
| --- | --- | --- | --- | --- | --- | --- | --- |
|  | **Cost** | **QALY** | **ICER** | **Cost** | **QALY** | **ICER** |  |
| **Age 30** |  |  |  |  |  |  |  |
| **No Intervention** | 54 449 | 19.9 |  | 46 119 | 20.0 |  |  |
| **Intervention** | 78 063 | 19.9 | 6 817 620 | 67 241 | 20.1 | 348 442 |  |
| **Age 50** |  |  |  |  |  |  |  |
| **No Intervention** | 65 331 | 14.5 |  | 59 724 | 15.1 |  |  |
| **Intervention** | 75 640 | 14.7 | 45 389 | 69 339 | 15.4 | 38 943 |  |
| **Age 70** |  |  |  |  |  |  |  |
| **No Intervention** | 44 350 | 7.4 |  | 47 343 | 8.3 |  |  |
| **Intervention** | 46 008 | 7.7 | 7 570 | 48 362 | 8.6 | 3 900 |  |

Table S2: Sensitivity analysis: Cost, QALY and ICER, deterministic, with 10% increase in cost in states

|  | **Male** | | | **Female** | | | |
| --- | --- | --- | --- | --- | --- | --- | --- |
|  | **Cost** | **QALY** | **ICER** | **Cost** | **QALY** | **ICER** |  |
| **Age 30** |  |  |  |  |  |  |  |
| **No Intervention** | 59 306 | 19.9 |  | 50 641 | 20.1 |  |  |
| **Intervention** | 61 912 | 20.5 | 4 338 | 53 081 | 20.7 | 4 035 |  |
| **Age 50** |  |  |  |  |  |  |  |
| **No Intervention** | 71 514 | 14.5 |  | 65 716 | 15.2 |  |  |
| **Intervention** | 74 351 | 14.9 | 6 449 | 68 466 | 15.6 | 5 984 |  |
| **Age 70** |  |  |  |  |  |  |  |
| **No Intervention** | 49 193 | 7.5 |  | 52 512 | 8.3 |  |  |
| **Intervention** | 51 398 | 7.7 | 9 688 | 54 847 | 8.6 | 9 188 |  |

Table S3: Sensitivity analysis: Cost, QALY and ICER, deterministic, with 10% decrease in cost in states

|  | **Male** | | | **Female** | | | |
| --- | --- | --- | --- | --- | --- | --- | --- |
|  | **Cost** | **QALY** | **ICER** | **Cost** | **QALY** | **ICER** |  |
| **Age 30** |  |  |  |  |  |  |  |
| **No Intervention** | 48 523 | 19.9 |  | 41 434 | 20.1 |  |  |
| **Intervention** | 50 854 | 20.5 | 3 881 | 43 629 | 20.7 | 3 631 |  |
| **Age 50** |  |  |  |  |  |  |  |
| **No Intervention** | 58 512 | 14.5 |  | 53 768 | 15.2 |  |  |
| **Intervention** | 61 030 | 14.9 | 5 726 | 56 216 | 15.6 | 5 328 |  |
| **Age 70** |  |  |  |  |  |  |  |
| **No Intervention** | 40 249 | 7.5 |  | 42 965 | 8.3 |  |  |
| **Intervention** | 42 239 | 7.7 | 8 742 | 45 066 | 8.6 | 8 268 |  |

Table S4: Sensitivity analysis: Cost, QALY and ICER, deterministic, with 10% increase in cost of intervention

|  | **Male** | | | **Female** | | | |
| --- | --- | --- | --- | --- | --- | --- | --- |
|  | **Cost** | **QALY** | **ICER** | **Cost** | **QALY** | **ICER** |  |
| **Age 30** |  |  |  |  |  |  |  |
| **No Intervention** | 53 915 | 19.9 |  | 46 037 | 20.1 |  |  |
| **Intervention** | 56 492 | 20.5 | 4 292 | 48 464 | 20.7 | 4 014 |  |
| **Age 50** |  |  |  |  |  |  |  |
| **No Intervention** | 65 013 | 14.5 |  | 59 742 | 15.2 |  |  |
| **Intervention** | 67 799 | 14.9 | 6 335 | 62 450 | 15.6 | 5 893 |  |
| **Age 70** |  |  |  |  |  |  |  |
| **No Intervention** | 44 721 | 7.5 |  | 47 739 | 8.3 |  |  |
| **Intervention** | 46 921 | 7.7 | 9 664 | 50 061 | 8.6 | 9 141 |  |

Table S5: Sensitivity analysis: Cost, QALY and ICER, deterministic, with 10% decrease in cost of intervention

|  | **Male** | | | **Female** | | | |
| --- | --- | --- | --- | --- | --- | --- | --- |
|  | **Cost** | **QALY** | **ICER** | **Cost** | **QALY** | **ICER** |  |
| **Age 30** |  |  |  |  |  |  |  |
| **No Intervention** | 53 915 | 19.9 |  | 46 037 | 20.1 |  |  |
| **Intervention** | 56 273 | 20.5 | 3 927 | 48 245 | 20.7 | 3 652 |  |
| **Age 50** |  |  |  |  |  |  |  |
| **No Intervention** | 65 013 | 14.5 |  | 59 742 | 15.2 |  |  |
| **Intervention** | 67 582 | 14.9 | 5 840 | 62 232 | 15.6 | 5 419 |  |
| **Age 70** |  |  |  |  |  |  |  |
| **No Intervention** | 44 721 | 7.5 |  | 47 739 | 8.3 |  |  |
| **Intervention** | 46 716 | 7.7 | 8 767 | 49 852 | 8.6 | 8 315 |  |

Table S6: Sensitivity analysis: Cost, QALY and ICER, deterministic, with assumed 14-year effectiveness

|  | **Male** | | | **Female** | | | |
| --- | --- | --- | --- | --- | --- | --- | --- |
|  | **Cost** | **QALY** | **ICER** | **Cost** | **QALY** | **ICER** |  |
| **Age 30** |  |  |  |  |  |  |  |
| **No Intervention** | 53 915 | 19.9 |  | 46 037 | 20.1 |  |  |
| **Intervention** | 56 074 | 20.5 | 3 569 | 48 134 | 20.7 | 3 447 |  |
| **Age 50** |  |  |  |  |  |  |  |
| **No Intervention** | 65 013 | 14.5 |  | 59 742 | 15.2 |  |  |
| **Intervention** | 67 339 | 14.9 | 5 240 | 62 025 | 15.6 | 4 923 |  |
| **Age 70** |  |  |  |  |  |  |  |
| **No Intervention** | 44 721 | 7.5 |  | 47 739 | 8.3 |  |  |
| **Intervention** | 46 851 | 7.7 | 8 590 | 49 805 | 8.6 | 8 076 |  |

Table S7: Sensitivity analysis: Cost, QALY and ICER, deterministic, with 3.3% change in weight loss

|  | **Male** | | | **Female** | | | |
| --- | --- | --- | --- | --- | --- | --- | --- |
|  | **Cost** | **QALY** | **ICER** | **Cost** | **QALY** | **ICER** |  |
| **Age 30** |  |  |  |  |  |  |  |
| **No Intervention** | 53 915 | 19.9 |  | 46 037 | 20.1 |  |  |
| **Intervention** | 56 427 | 20.5 | 4 189 | 48 393 | 20.7 | 3 901 |  |
| **Age 50** |  |  |  |  |  |  |  |
| **No Intervention** | 65 013 | 14.5 |  | 59 742 | 15.2 |  |  |
| **Intervention** | 67 740 | 14.9 | 6 215 | 62 386 | 15.6 | 5 767 |  |
| **Age 70** |  |  |  |  |  |  |  |
| **No Intervention** | 44 721 | 7.5 |  | 47 739 | 8.3 |  |  |
| **Intervention** | 46 851 | 7.7 | 9 387 | 49 990 | 8.6 | 8 884 |  |

Table S8: Sensitivity analysis: Cost, QALY and ICER, deterministic, with 3.3% change in weight loss only

|  | **Male** | | | **Female** | | | |
| --- | --- | --- | --- | --- | --- | --- | --- |
|  | **Cost** | **QALY** | **ICER** | **Cost** | **QALY** | **ICER** |  |
| **Age 30** |  |  |  |  |  |  |  |
| **No Intervention** | 53 915 | 19.9 |  | 46 037 | 20.1 |  |  |
| **Intervention** | 56 542 | 20.5 | 4 392 | 48 440 | 20.7 | 3 985 |  |
| **Age 50** |  |  |  |  |  |  |  |
| **No Intervention** | 65 013 | 14.5 |  | 59 742 | 15.2 |  |  |
| **Intervention** | 67 959 | 14.9 | 6 744 | 62 539 | 15.6 | 6 127 |  |
| **Age 70** |  |  |  |  |  |  |  |
| **No Intervention** | 44 721 | 7.5 |  | 47 739 | 8.3 |  |  |
| **Intervention** | 47 027 | 7.7 | 10 230 | 50 160 | 8.6 | 9 621 |  |

Figure S1: Cost-effectiveness planes by age (younger (30), middle (50), older (70)) and sex (male, female), 1 000 simulations, when transition from type 2 diabetes to any pre-diabetic states was not possible

|  | **Male** | **Female** |
| --- | --- | --- |
| **30** | **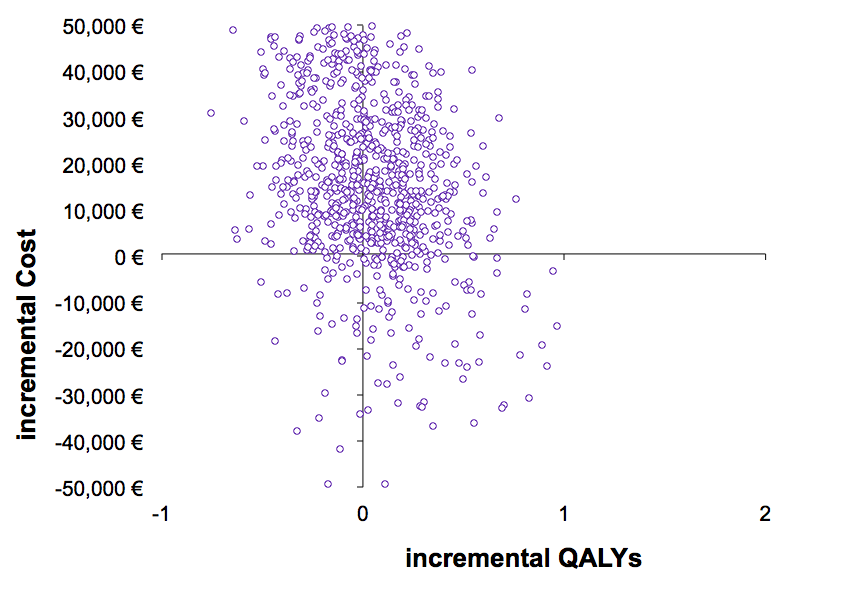** | **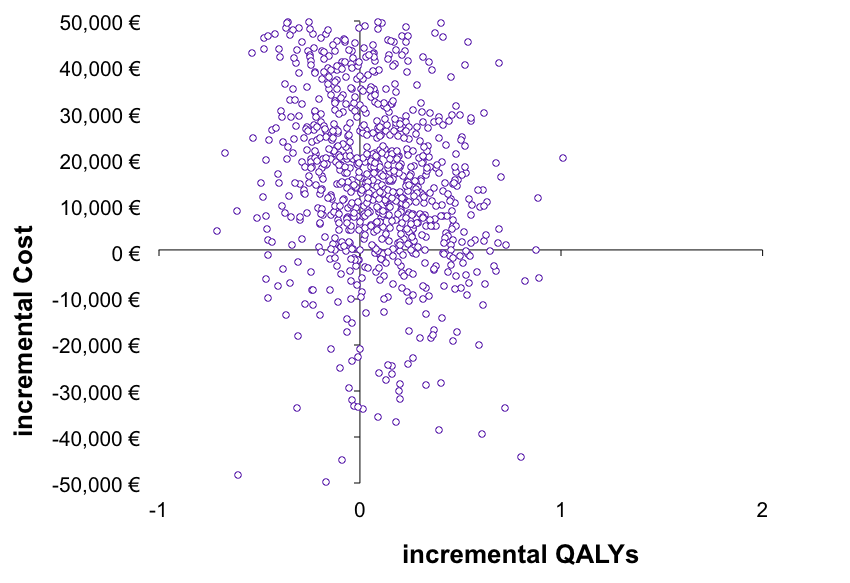** |
| **50** | 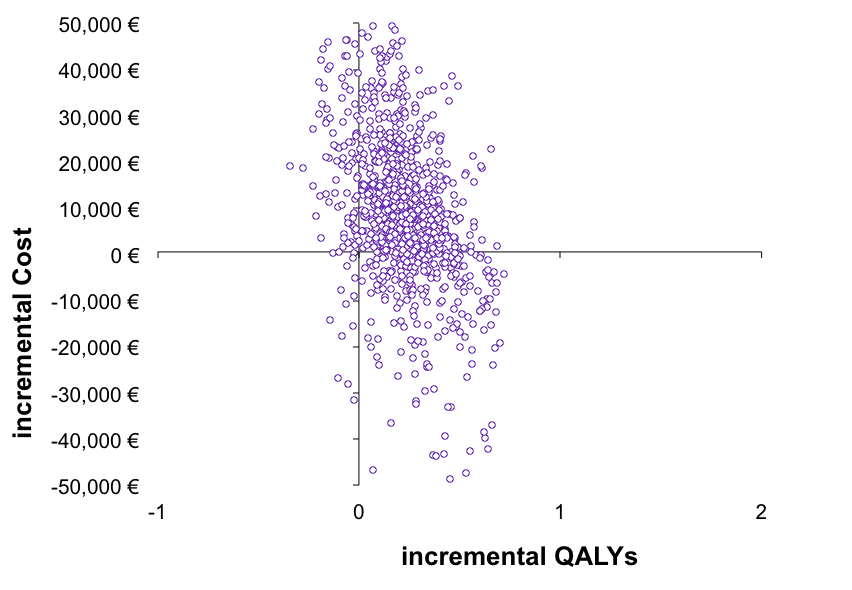 | 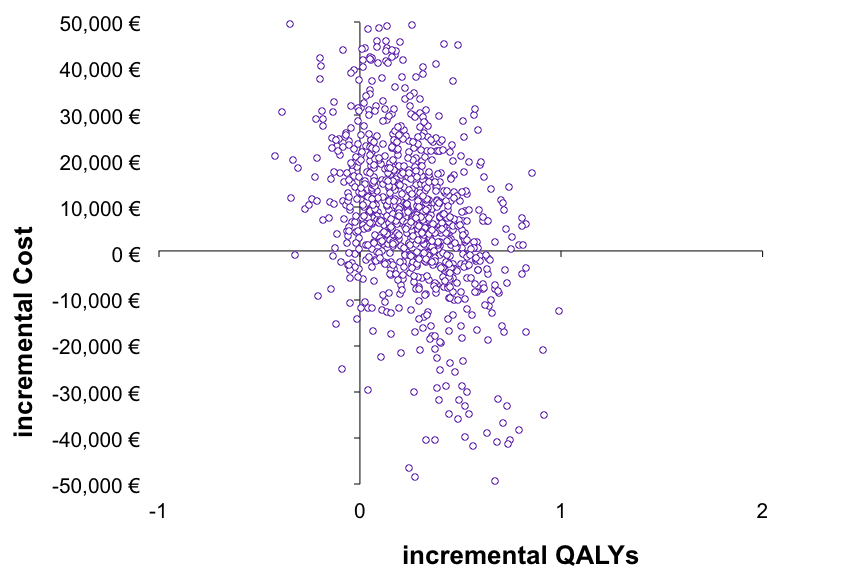 |
| **70** | 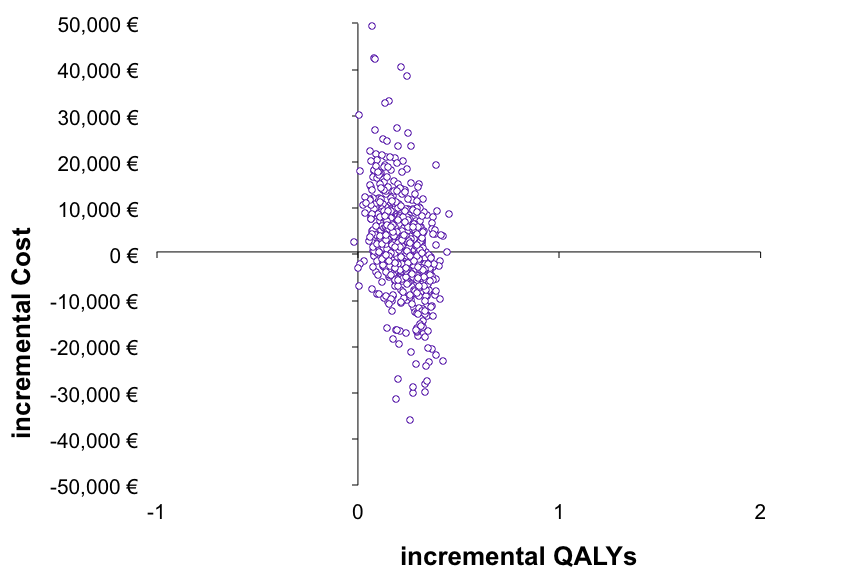 | 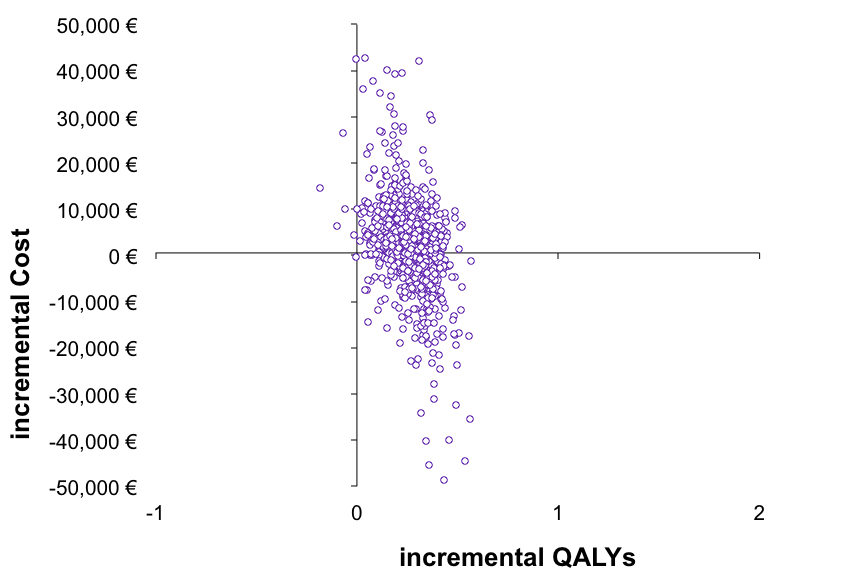 |

Figure S2: Cost-effectiveness acceptability curves by age (younger (30), middle (50), older (70)) and sex (male, female), 1 000 simulations, with 3.3% change in weight loss

|  | **Male** | **Female** |
| --- | --- | --- |
| **30** | **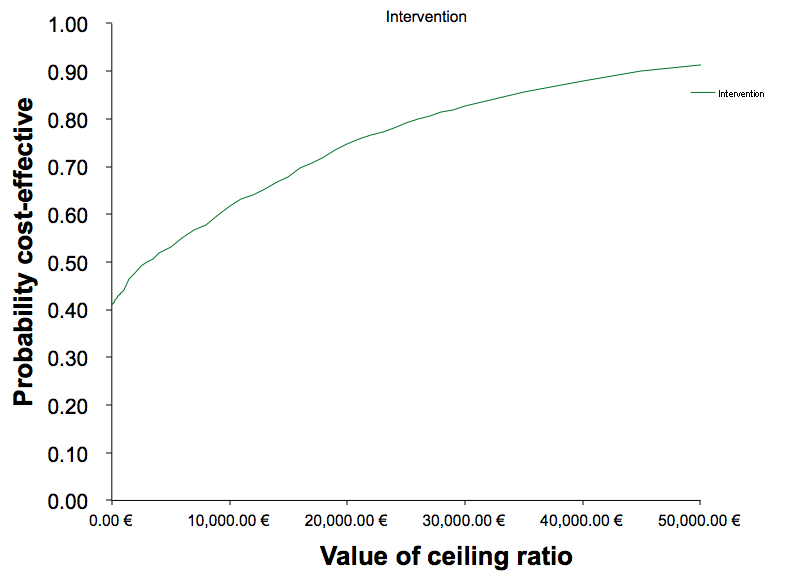** | **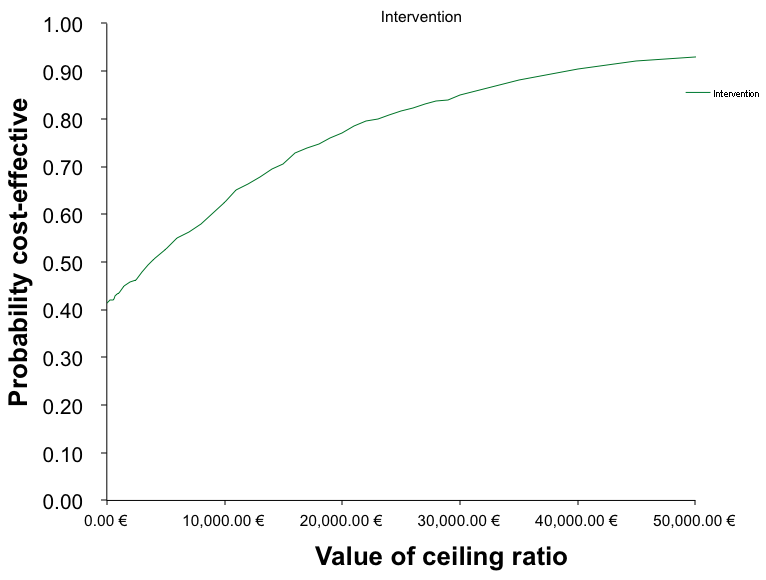** |
| **50** | 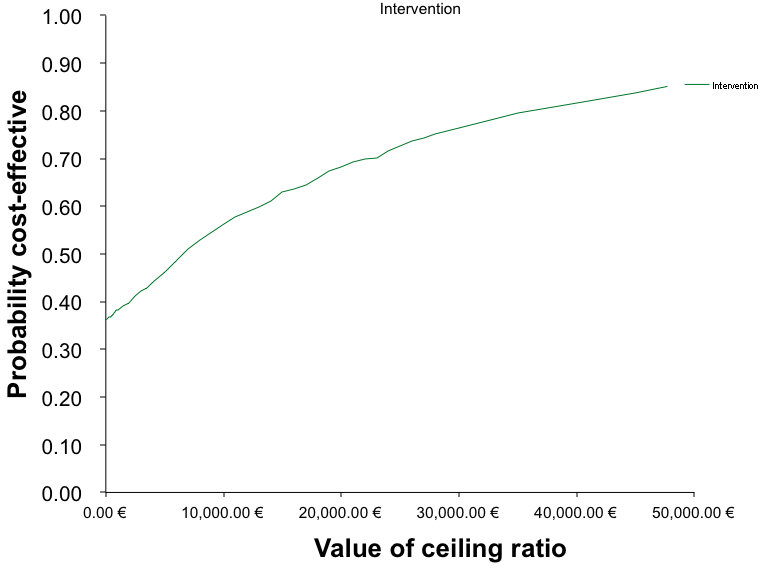 | 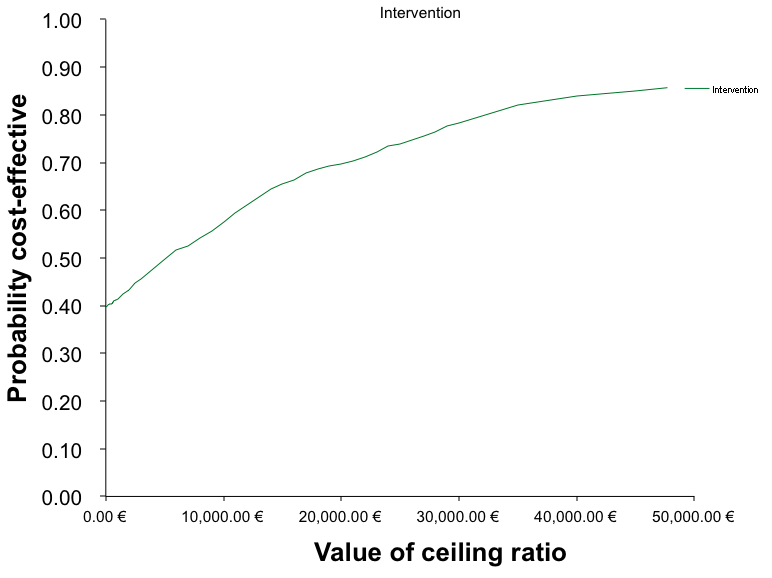 |
| **70** | 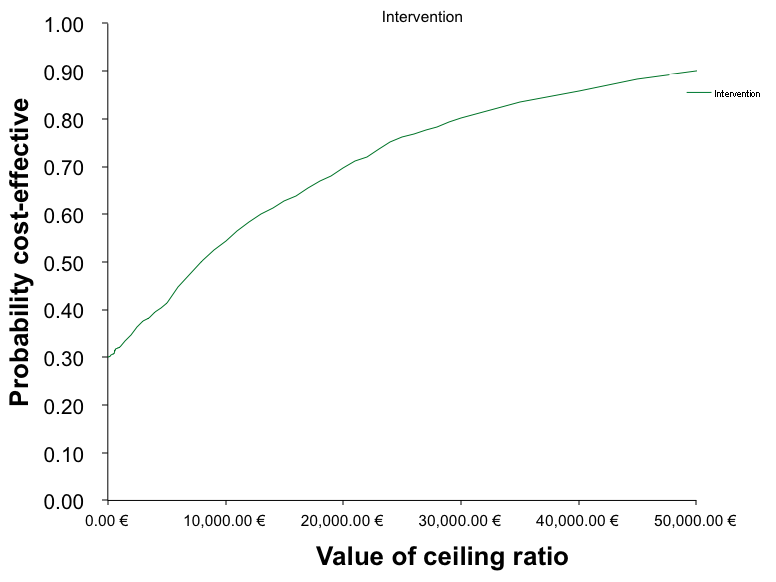 | 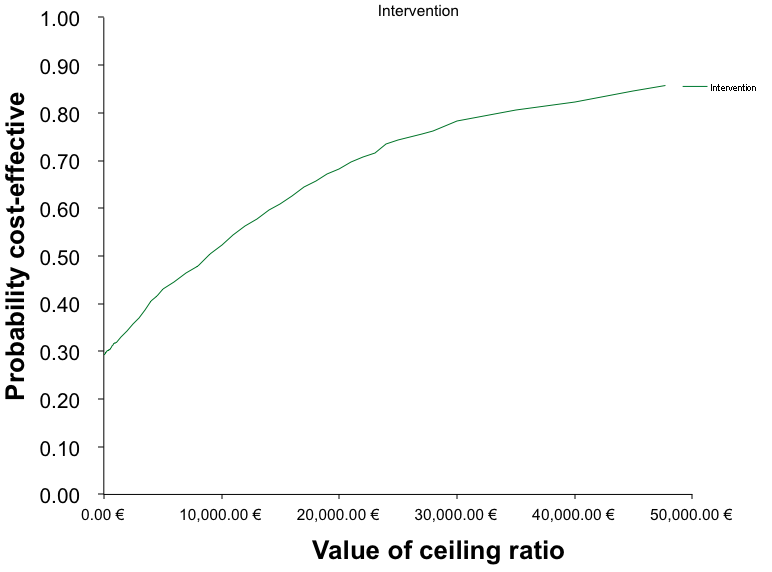 |

Figure S3: Cost-effectiveness acceptability curves by age (younger (30), middle (50), older (70)) and sex (male, female), 1 000 simulations, with 3.3% change in weight loss only

|  | **Male** | **Female** |
| --- | --- | --- |
| **30** | **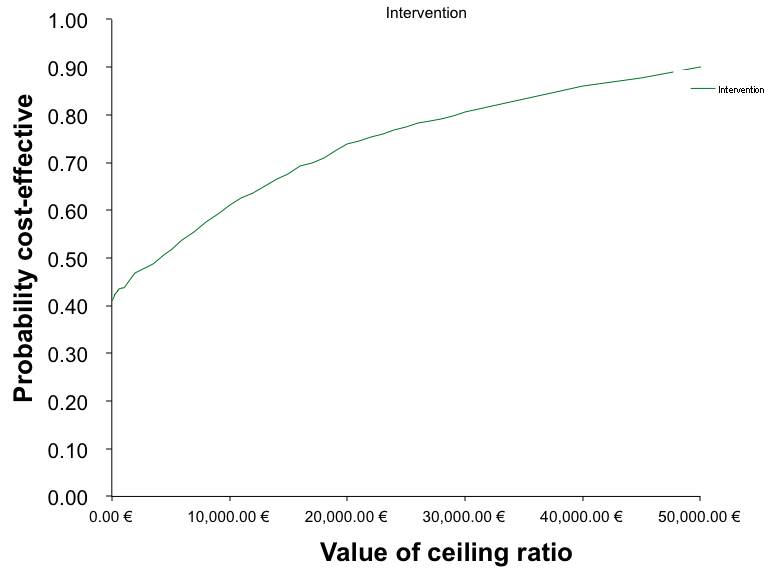** | **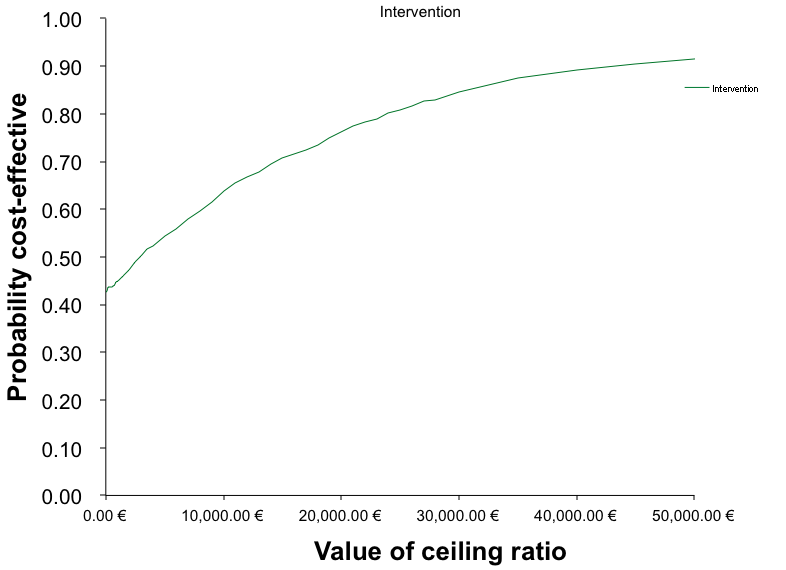** |
| **50** | 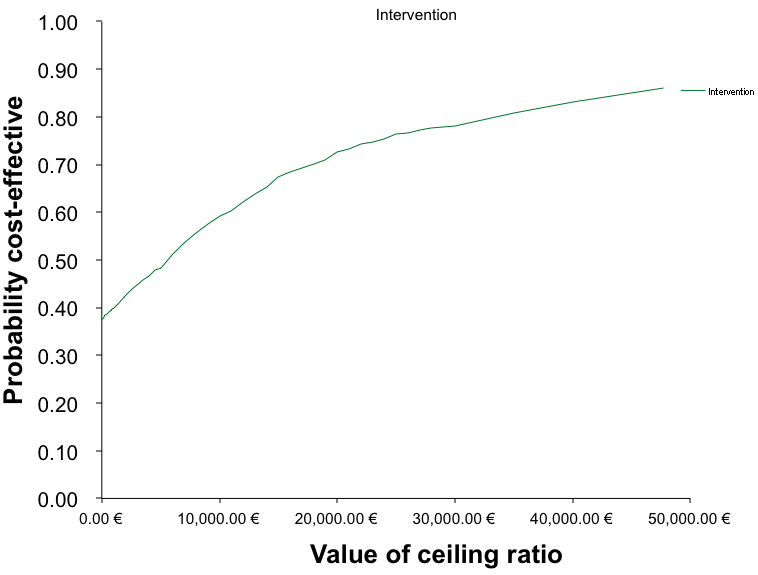 | 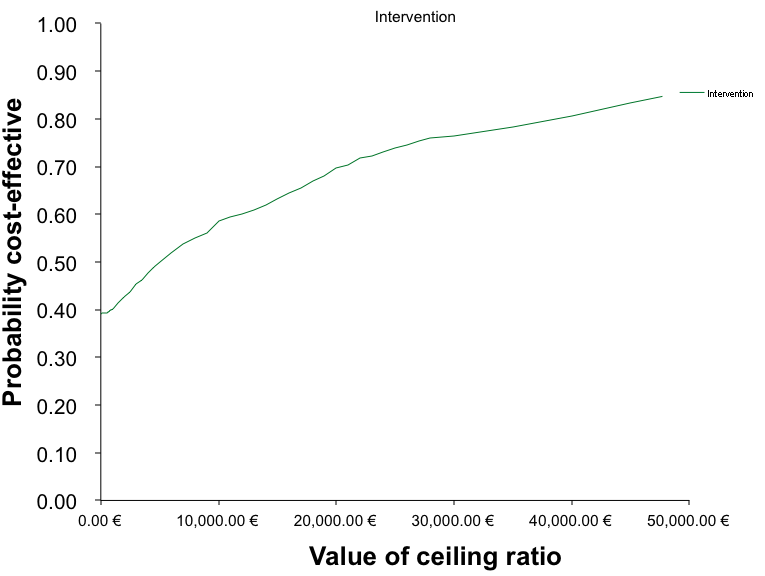 |
| **70** | 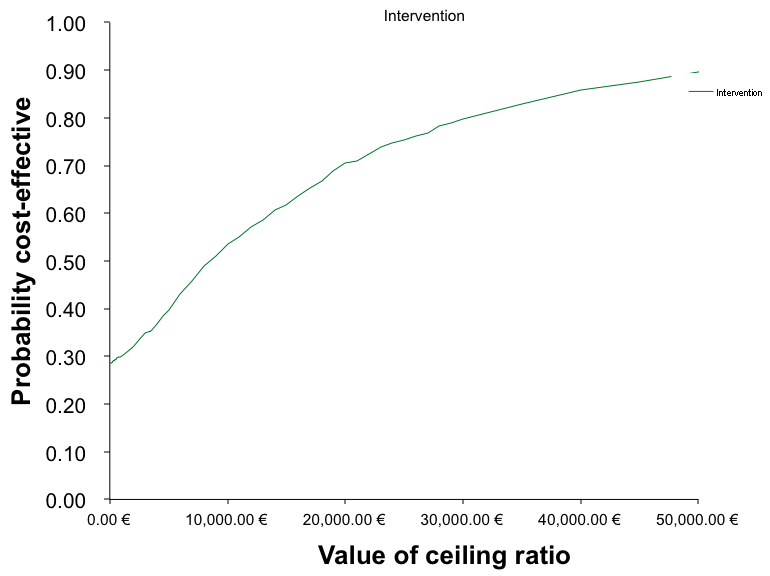 | 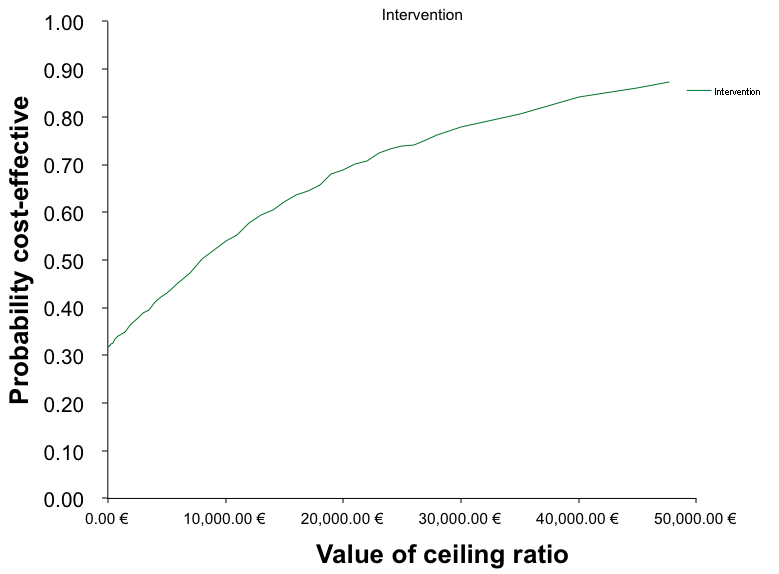 |
